# Gene silencing of *Helicobacter pylori* through newly designed siRNA convenes the treatment of gastric cancer

**DOI:** 10.1002/cam4.6772

**Published:** 2023-12-01

**Authors:** Mahjerin Nasrin Reza, Shahin Mahmud, Nadim Ferdous, Ishtiaque Ahammad, Mohammad Uzzal Hossain, Md. Al Amin, A. K. M. Mohiuddin

**Affiliations:** ^1^ Department of Biotechnology and Genetic Engineering, Life Science Faculty Mawlana Bhashani Science and Technology University Tangail Bangladesh; ^2^ Bioinformatics Division National Institute of Biotechnology Ashulia Bangladesh

**Keywords:** CagA and VacA, gastric cancer, *Helicobacter pylori*, siRNA

## Abstract

**Background:**

*Helicobacter pylori* is a gastric pathogen that is responsible for causing chronic inflammation and increasing the risk of gastric cancer development. It is capable of persisting for decades in the harsh gastric environment because of the inability of the host to eradicate the infection. Several treatment strategies have been developed against this bacterium using different antibiotics. But the effectiveness of treating *H. pylori* has significantly decreased due to widespread antibiotic resistance, including an increased risk of gastric cancer. The small interfering RNAs (siRNA), which is capable of sequence‐specific gene‐silencing can be used as a new therapeutic approach for the treatment of a variety of such malignancies. In the current study, we rationally designed two siRNA molecules to silence the cytotoxin‐associated gene A (CagA) and vacuolating cytotoxin A (VacA) genes of *H. pylori* for their significant involvement in developing cancer.

**Methods:**

We selected a common region of all the available transcripts from different countries of CagA and VacA to design the siRNA molecules. The final siRNA candidate was selected based on the results from machine learning algorithms, off‐target similarity, and various thermodynamic properties.

**Result:**

Further, we utilized molecular docking and all atom molecular dynamics (MD) simulations to assess the binding interactions of the designed siRNAs with the major components of the RNA‐induced silencing complex (RISC) and results revealed the ability of the designed siRNAs to interact with the proteins of RISC complex in comparable to those of the experimentally reported siRNAs.

**Conclusion:**

These designed siRNAs should effectively silence the CagA and VacA genes of *H. pylori* during siRNA mediated treatment in gastric cancer.

## 1 INTRODUCTION

The gram‐negative bacterium *Helicobacter pylori* specifically colonizes the gastric epithelium.^1^ The spiral‐shaped bacterium has 3–5 polar flagella that are utilized for locomotion and is positive for the urease, catalase, and oxidase enzymes.^2^ Most *H. pylori* strains express virulence factors that have evolved it interfere with host cell signalling pathways, this remarkable ability to persist for decades in the harsh stomach environments, primarily because the host cannot effectively eliminate it.^3^, ^4^ By utilizing urease to convert urea into ammonia, *H. pylori* has acquired the ability to thrive in the extremely acidic environment, setting it apart from other viruses and bacteria.^5^ For many years, experts disagreed as to whether *H. pylori* causes stomach cancer. But other studies, including one involving 1526 Japanese patients, have now clearly demonstrated that *H. pylori* infection greatly raises the risk of stomach cancer.^6^ Uemura et al. reported that about 3% of people with *H. pylori* infection had gastric cancer, whereas the uninfected patients had no sign of it.^6^ Individuals infected with *H. pylori* but without premalignant lesions had a significantly reduced risk of developing stomach cancer following *H. pylori* eradication. Randomized prospective studies have shown a substantial decreases in the presence of premalignant lesions after eradication, highlighting the role of this organism in early gastric carcinogenesis.^7^, ^8^

The small interfering RNAs (siRNAs), that are capable of regulating gene expression through a procedure named RNA interference (RNAi), have been regarded as one of the most notable advances.^9^ Since their discovery in the 1990s, RNAi therapeutics have shown great potential for reducing the expression of disease‐related genes.^10^ A significant milestone for RNAi therapy was achieved in 2018 when the first siRNA‐based drug ‘Patisiran’ (Onpattro®) received approval for the treatment of transthyretin‐mediated amyloidosis.^11^ In this therapeutic method, the double‐stranded RNAs (dsRNA) designed to target disease‐causing mRNA sequences are incorporated into a gene regulatory complex known as the RNA‐induced silencing complex (RISC), consisting of DICER, Argonaute‐2 (Ago2), and transactivation response RNA‐binding protein (TRBP).^12^, ^13^ The enzyme Dicer first initiates the RNAi mechanism by splitting double‐stranded RNAs (dsRNAs) into 21–25 nt long, double‐stranded siRNAs. The siRNA guide strand is then filled onto the RNA‐induced silencing (RISC) complex and the siRNA passenger strand is unwound.^9^ As a result, the target mRNAs can be cleaved by Argonaute 2 (Ago2). When the guide strand sequence is coupled with an mRNA corresponding sequence.^9^

The vast majority of earlier research on siRNA design is sequence specific to a target gene. Sohrab et al. designed potential siRNAs targeting ORF1ab of MERS‐CoV and experimentally validated them in Vero cell line.^14^ Oany et al. designed a most probable siRNA in order to repress the nucleocapsid gene of several Nipah virus strains.^15^ Chowdhury et al. designed several siRNA molecules targeting the nucleocapsid phosphoprotein and surface glycoprotein gene of SARS‐CoV‐2 and showed the synergy of these siRNAs with Ago2 protein.^16^ To effectively address these maladies, it is essential to employ a proper computational approach for siRNA design and development.^17^ Additionally, the issues of stability and early in vivo clearance are resolved by incorporating siRNA molecules onto specialized nanocarrier tailored for specific tissues or cells.^18^ These nanocarriers enhance the overall effectiveness of naked siRNA molecules by mitigating concerns such as off‐target binding and undesired immune reactions.^19^

**FIGURE 1:**
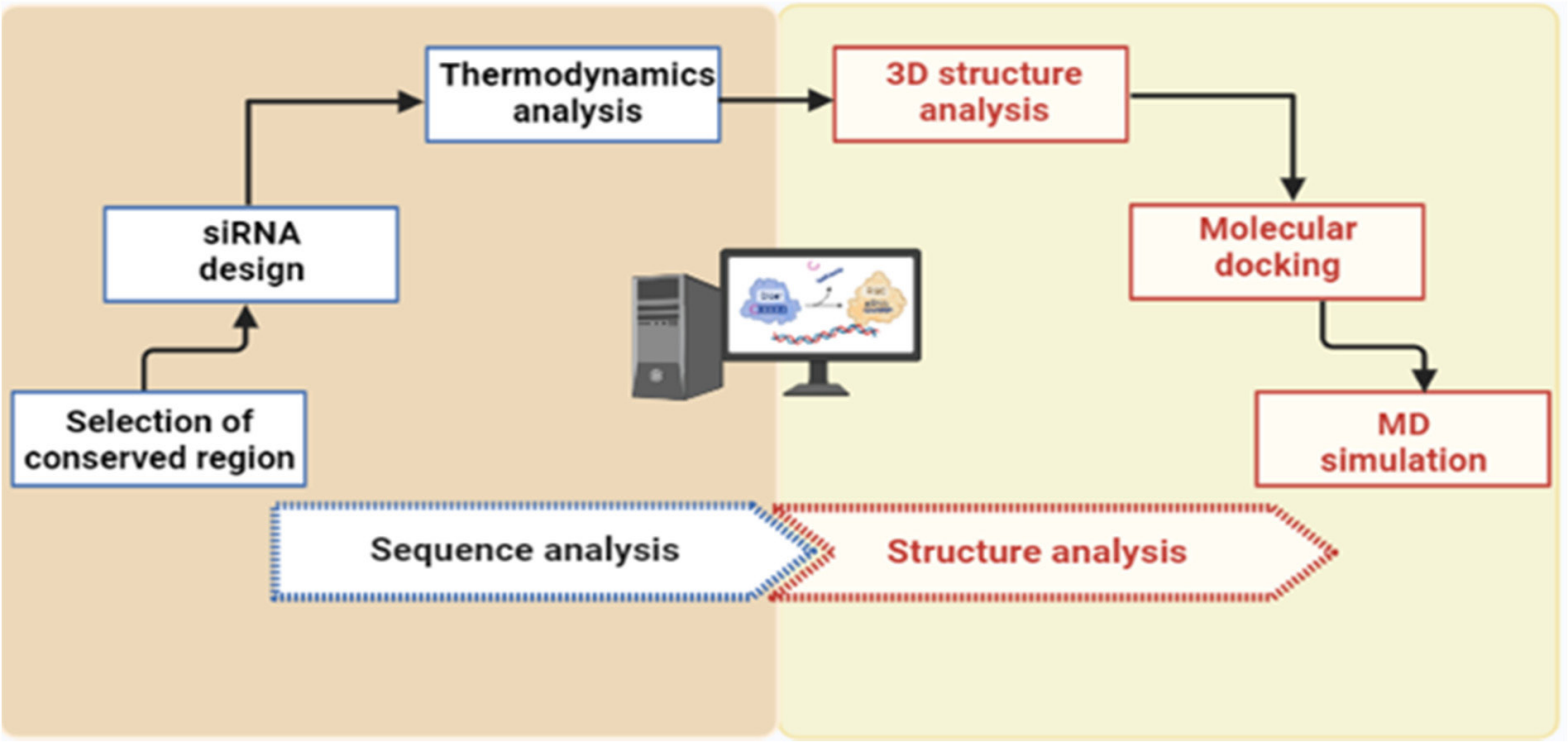
The overall summary of this study.

Therefore, in this study, we aimed to utilize several computational algorithms and molecular dynamics (MD) simulations to design a potential siRNA targeting the CagA and VacA genes of *H. pylori* which will suppress the translations of these virulent proteins, allowing the host to eradicate this infection. siRNAs were designed against both CagA and VacA as both are abundant proteins produced by the bacterium and targeting these proteins might result in bacterial inhibition. We hope this study will help to develop a new treatment strategy for *H. pylori* mediated gastric cancer. The overall workflow of the study including methodological steps is shown in Figure 1.

## 2 MATERIALS AND METHODS

### 2.1 Pathophysiology of *H. pylori* and targeted gene identification

*H. pylori* is the primary risk factor for this cancer. Targeting *H. pylori*‐infected individuals at high risk for stomach cancer for management is necessary.^20^ In *H. pylori* most well‐known genes are cytotoxin‐associated gene A (CagA) and cytotoxin gene (VacA).^21^ The severe form of gastroduodenal disease is caused worldwide by the virulence factors cagA and vacA genotypes and their variations.^22^ Several study cytotoxin‐associated gene A (CagA) and cytotoxin gene (VacA) demonstrated, exhibit various signature pathways of initiation of gastric adenocarcinoma (GAC).^23^ Besides, *H. pylori* exhibits varying levels of antibiotic resistance in various geographic regions; this is one of the primary causes for the absence of treatment.^24^ We explored CagA and VacA gene powerful virulence factors for GAC and designed siRNA against this pathogenicity for lowering the infection in human.

### 2.2 Selection of cDNA sequences of target gene

We collected CagA cDNA sequences from seven countries (Bangladesh, Sweden, USA, Australia, China, Philippines, and Vietnam) and VacA cDNA sequences from six countries (Bangladesh, Nepal, Japan, Indonesia, India, and China) respectively from NCBI database (https://www.ncbi.nlm.nih.gov/). As there were no available CagA and VacA cDNA sequences of Bangladesh in NCBI database, we collected the available *H. pylori* whole genome sequences from this country. In case of other selected countries, the available CagA and VacA cDNA sequences were collected and subjected to multiple sequence alignment (MSA) using Clustal Omega^25^ to find the conserved regions of the two genes in these sequences.

### 2.3 Prediction of computational designed siRNAs

The conserved sequences were used to predict the possible siRNAs against the VacA and CagA genes. In this regard, the i‐SCORE Designer web tool (https://www.med.nagoya‐u.ac.jp/neurogenetics/i_Score/i_score.html) was utilized for sequence‐based siRNA design.^26^ This program analyzes several target mRNA nucleotide preferences to generate nine alternative algorithm scores (Ui‐Tei, Amarzguioui, Hsieh, Takasaki, s‐Biopredsi, i‐Score, Reynolds, Ka toh, and DSIR) for siRNA prediction. Depending on their calculating nature, sequence‐based algorithms can also be classified into two groups: rule‐based and machine‐learning aided. For this research, rule‐based approaches such as the Ui‐Tei, Amarzguioui, and Reynolds scoring systems were brought into consideration. For machine‐learning aided siRNA prediction, the i‐Score (inhibitory score) algorithm was used, that employs a linear regression model to forecast siRNAs. This method solely looks at the nucleotide preferences at each location when calculating the i‐score. We only selected the results for each of the five algorithms that scored at or above the stated cutoff values.

### 2.4 Filtration of off‐target sites

A filtration approach was used to exclude the possibility of producing off‐target effects of the potential siRNAs. The human RefSeq mRNA database was evaluated for a perfect (19/19) or near‐perfect (18/19, 17/19) match using nucleotide BLAST (https://blast.ncbi.nlm.nih.gov/Blast.cgi). The screening was performed against both sense and antisense strands of candidate siRNAs. The siRNAs that showed complete or nearly complete complementarity with off‐target mRNA were excluded considering that they would induce off‐target effects.

### 2.5 Thermodynamic analysis

The OligoEvaluator analysis tool (http://www.oligoevaluator.com) was used to determine the internal melting temperature (Tm) of the sense strand of each possible siRNA duplex. The MaxExpect tool^27^ of the RNA structure website was used to predict the siRNA secondary structure along with the corresponding free energy. Higher energy values depict better candidates because those molecules are less likely to fold. Also, higher target‐guide strand interaction indicates better siRNA efficacy. The DuplexFold tool^28^ of the RNA structure web server was utilized to predict the target strand and the siRNA guide strand's thermodynamic interaction.

### 2.6 3D structural modelling of siRNAs

We generated the 3D structure of the chosen siRNAs using the freely available RNAComposer server^29^ (http://rnacomposer.ibch.poznan.pl/Home). This server utilizes the RNA FRABASE database, a search engine compatible with the RNA tertiary structures database. To forecast complex structures like multi‐branched loops and pseudo‐knotted loops, a motif template‐based technique is also used. The RNAalifold web server^30^ (http://rna.tbi.univie.ac.at/cgi‐bin/RNAWebSuite/RNAalifold.cgi) predicted the secondary structure of the siRNAs in the dot‐bracket notation (Vienna format) which was used as the input for the RNA composer web tool. The dot sign used in dot‐bracket notation indicates the site of unpaired nucleotide. Finally, the 3D structure of the siRNAs was downloaded in PDB (Protein Data Bank) file format.

### 2.7 Binding affinity of designed siRNAs with the components of RISC‐Loading complex

We performed a series of molecular docking of designed siRNAs with transactivation response element RNA‐binding protein (TRBP), dicer, and argonaute‐2 proteins, major components of RISC‐Loading complex. The crystal structures of these three proteins (5N8L, 4NGF, and 6RA4) were available in the Protein Data Bank^31^ (https://www.rcsb.org/). The HDOCK server^32^ was used for this purpose (http://hdock.phys.hust.edu.cn/). The HDOCK server accepts amino acid sequences as input and makes use of a hybrid docking method that enables the incorporation of experimental information on the protein–protein binding site and small‐angle X‐ray scattering throughout the docking and post‐docking processes. Moreover, HDOCK has an intrinsic scoring function that enables protein‐RNA/DNA docking. The RNA‐protein interactions were analyzed by the BIOVIA Discovery Studio tool.^33^

### 2.8 Molecular dynamics (MD) simulation of siRNAs and siRNA‐protein complexes

The designed siRNAs, siRNA‐protein complexes, and relevant controls were subjected to MD simulation using the CHARMM36 force field, which was produced with the CHARMM‐GUI server and run with the GROMACS 2018.3 program.^34^ The solvation of the complexes was done using a rectangular box with a padding distance of 1.0 nm. Then, the solvated systems were neutralized by the addition of the counter ions. Moreover, the steepest descent algorithm was used to minimize the energy of the system. A 100 ps equilibration under NVT ensemble was performed after energy minimization. The systems were equilibrated using NPT ensemble for 1 ns in the following phase. The same molecular dynamic approach was used for the simulation of siRNAs in their final state for up to 100 ns. All the simulations were performed at 300 K temperature with 1 atm pressure for mimicking the general experimental conditions and a 2 fs time steps. GROMACS functions were used to evaluate the hydrogen bonds, radius of gyration (Rg), and root means square deviation (RMSD). Molecular dynamics simulations and result evaluations were done in the high‐performance computing (HPC) cluster of the Bioinformatics Division at National Institute of Biotechnology, Bangladesh.

## 3 RESULTS

### 3.1 Sequence retrieval of targeted gene and siRNA design

**FIGURE 2:**
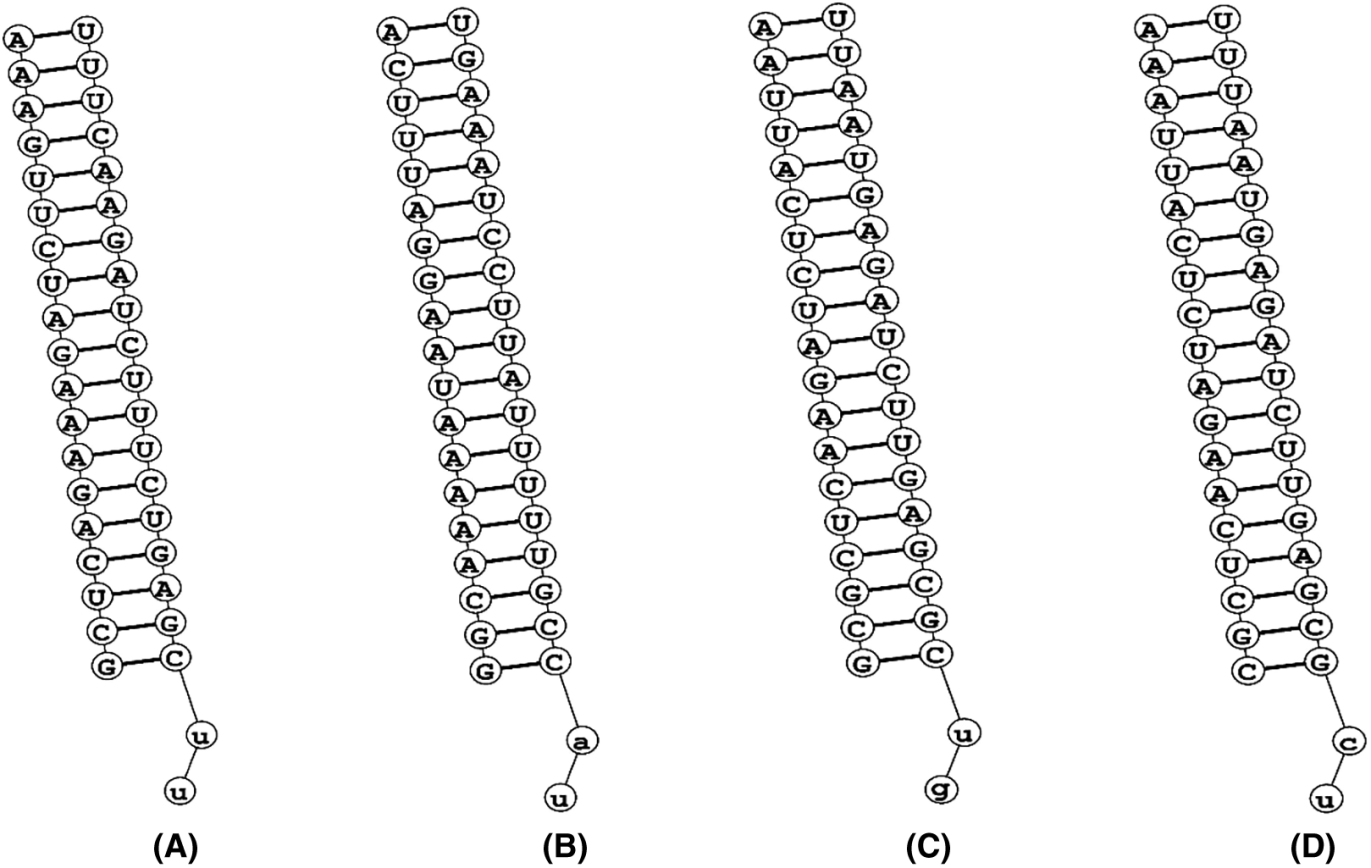
The two‐dimensional representation of the four siRNAs that have been designed. For off‐target filtration, BLAST analysis was performed with both strands of candidate siRNAs against the human genome to filter out the undesired siRNAs. It was found that none of the selected siRNAs possessed nearly identical sequence segments except the transcripts of the two genes.

Two potential siRNAs were identified for each gene after submitting the conserved regions into the i‐SCORE Designer program and those siRNAs passed the suggested cutoff value for each of the five algorithms listed above. The percentage of GC in each siRNAs was observed since a low GC content can lead to poor and non‐specific binding, while a high GC content prevents the RISC complex (RNA‐Induced Silencing Complex) and helicase from unfolding the siRNA duplex. Various acceptable GC content thresholds have been proposed by numerous researches. In our current study, we have designed two unique siRNAs, Table 1 is highlighting a comprehensive overview of their corresponding i‐scores, amarzguioui, and GC content. Additionally, Figure 2 is representing the two‐dimensional structures of the selected siRNAs, offering a visual of their probable binding nature. According to,^35^ it is advisable for GC content of siRNAs to span from 30% to 60%, considering all nucleotide preferences. Notably, our designed siRNA demonstrated the GC content of all the selected siRNAs aligns with this specified range.

**TABLE 1:** Two selected siRNAs from two genes including the sequence of sense and antisense strands, percentage of GC content, scores from different rule‐based methods, and the i‐Scores.

Given name	Sense	Antisense	%GC	Ui‐Tei (Ia/Ib)	Amarzguioui (> = 3)	Reynolds (> = 6)	i‐Score (> = 66)
siRNA_CagA‐1	GCUCAGAAAGAUCUUGAAA	UUUCAAGAUCUUUCUGAGCuu	36.8	Ia	4	7	72.8
siRNA_CagA‐2	GGCAAAAAUAAGGAUUUCA	UGAAAUCCUUAUUUUUGCCau	31.6	Ia	5	6	72.2
siRNA_VacA‐1	GCGCUCAAGAUCUCAUUAA	UUAAUGAGAUCUUGAGCGCug	42.1	Ia	5	8	72.6
siRNA_VacA‐2	CGCUCAAGAUCUCAUUAAA	UUUAAUGAGAUCUUGAGCGcu	36.8	Ia	6	9	84.4

### 3.2 Thermodynamics of guide and target‐guide strand interaction

**FIGURE 3:**
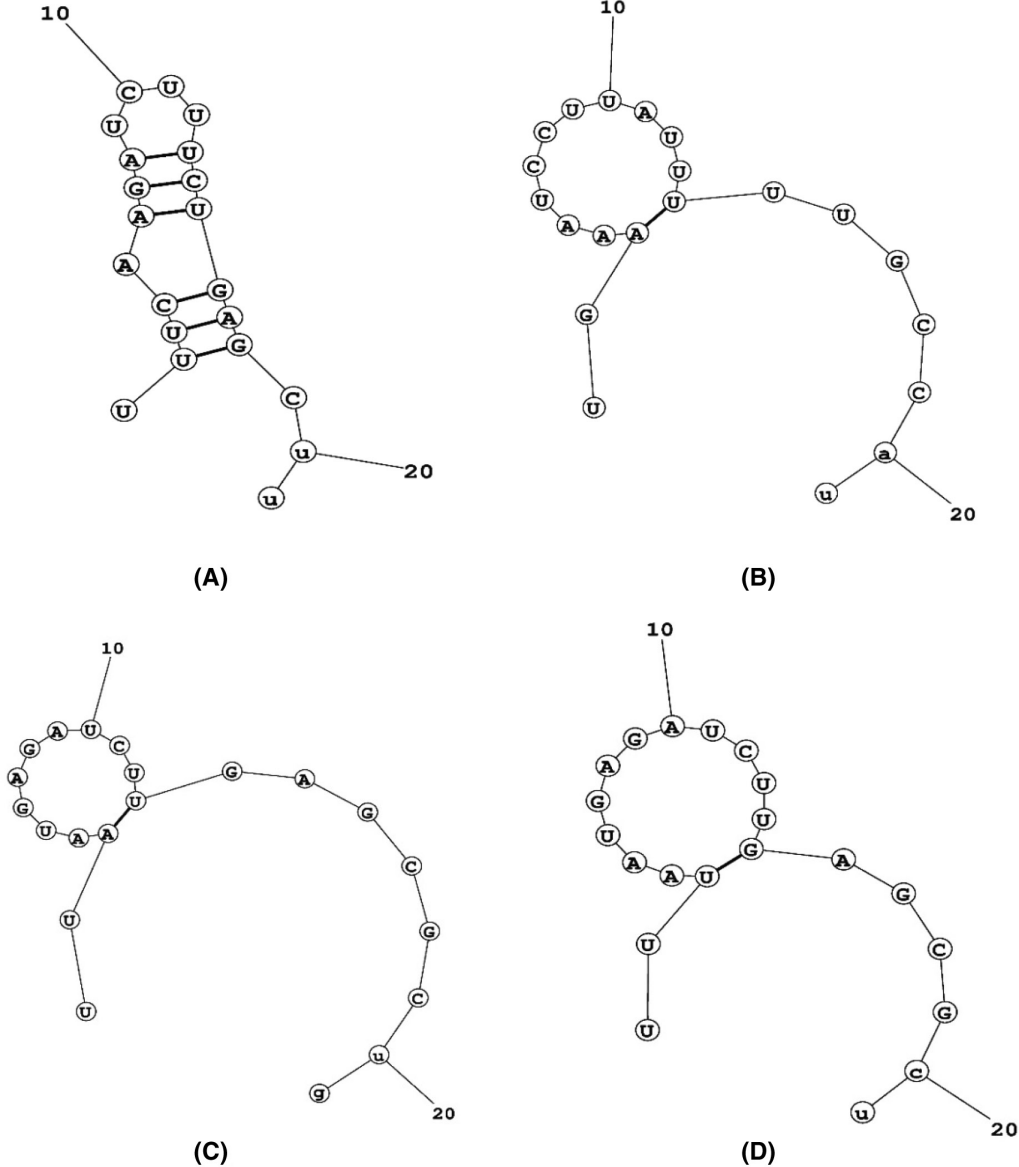
Secondary structures of the designed siRNAs with probable folding and lowest free energy for consensus sequence.

The thermodynamic stability of nucleotide base pairing plays a significant role in modulating the silencing mechanism of siRNA (Figure 3). The internal melting temperature (Tm) and free energy change (ΔG) between siRNA seed and mRNA target are trustworthy markers of the thermodynamic stability of such heteroduplexes. All four siRNAs were found to have internal melting temperatures (Tm) below 65°C. The calculated free energy of folding of the guide strands ranged from 1.6 to 1.8 for the four siRNAs. Additionally, the associated secondary structures were also identified. The free energy of binding between targets and guide strand was calculated. The values spanned from −28 to −32. Based on this analysis, we selected the siRNA_CagA‐1 and the siRNA_VacA‐1 for further analysis.

### 3.3 3D structure of the selected siRNAs

**FIGURE 4:**
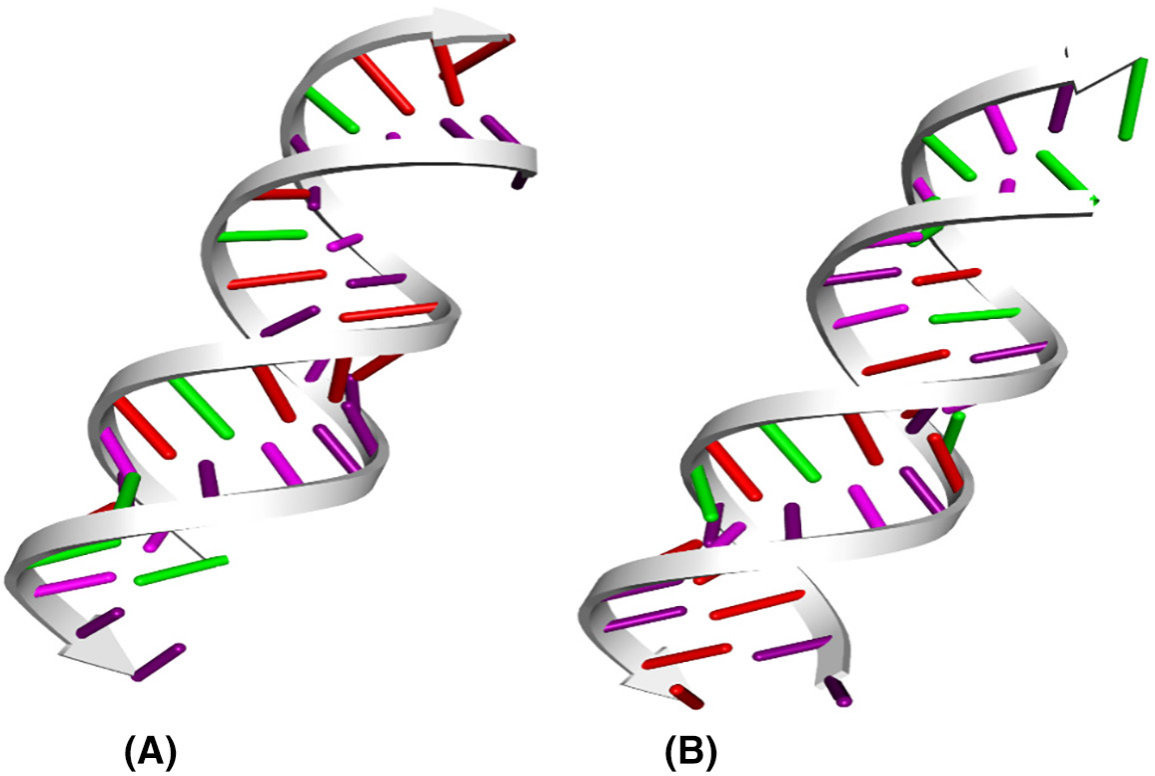
3D structures of the designed siRNAs: (A) siRNA_CagA‐1 and (B) siRNA_VacA‐1.

To visualize the intricate 3D structure of the chosen siRNAs, we utilized the RNAComposer server (Figure 4). This multifaceted process unfolded through a series of steps, commencing with secondary structure fragmentation, 3D structure elements preparation, rigid body transformation, and optimizing their arrangement for precision and accuracy. It provided the output model in protein data bank (PDB) file format.

### 3.4 Molecular docking results

**FIGURE 5:**
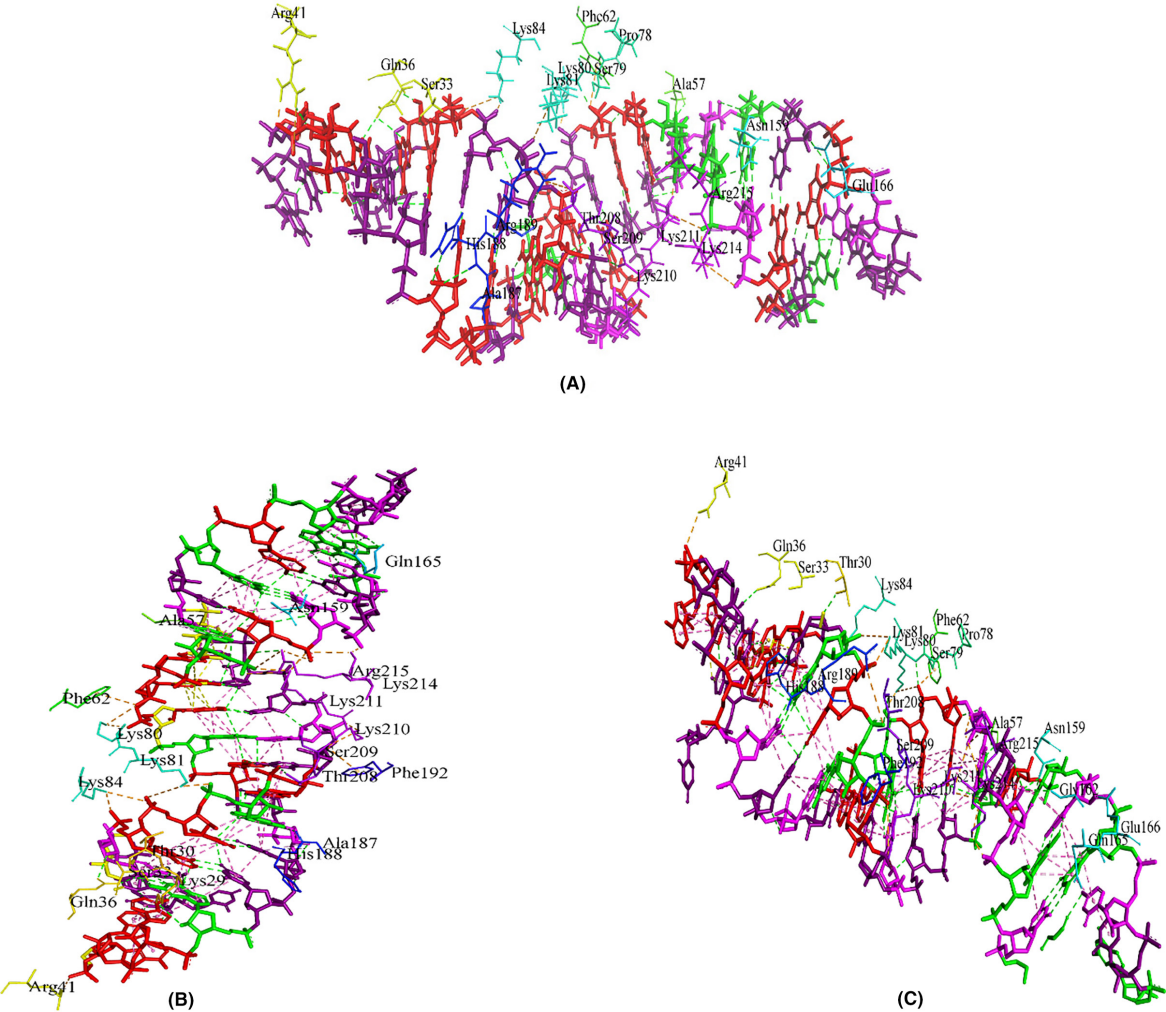
Protein‐siRNA interactions between (A) human Dicer and control, (B) siRNA_CagA and (C) siRNA_VacA.

We performed a series of molecular docking of our designed siRNAs with the major components of the RISC‐Loading complex. First, we performed molecular docking of two designed siRNAs with human dicer. The crystal structure of the human Dicer Platform‐PAZ‐Connector Helix cassette in complex with 17‐mer siRNA (PDB ID: 4NGF) is available in the RCSB PDB database. We identified the binding residues of the human dicer with the control siRNA and docked our designed siRNAs targeting those residues of the structure. We found a number of 10 common interactions between dicer‐designed_siRNA (CagA) and the control and found a number of 9 common interactions between dicer‐designed_siRNA (VacA) and the control. The interacting residues of control siRNA, designed siRNAs against CagA and VacA with dicer are shown in Figure 5.

**FIGURE 6:**
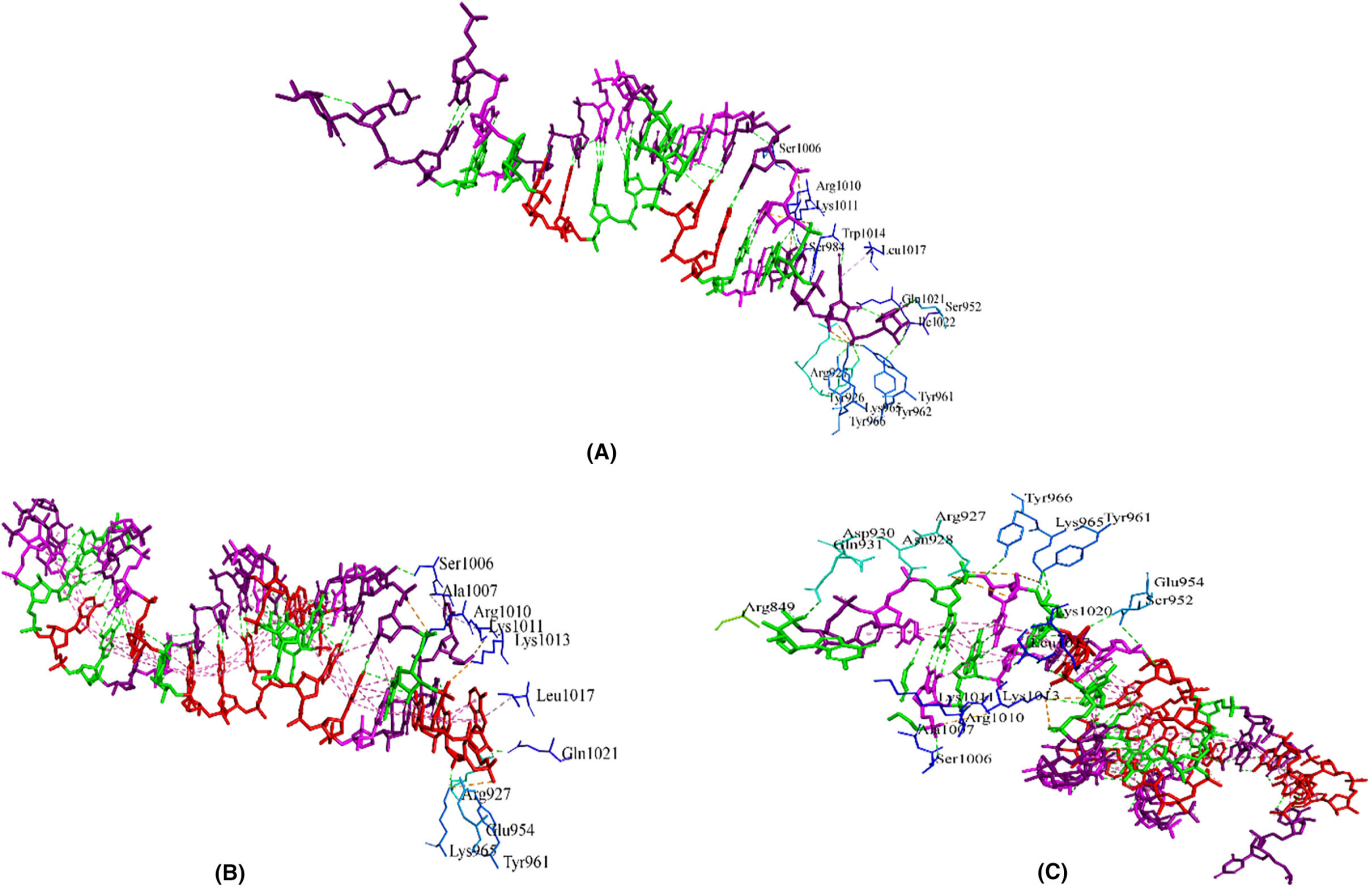
Protein‐siRNA interactions between (A) TRBP and control, (B) siRNA_CagA and (C) siRNA_VacA.

Then, we performed molecular docking of two designed siRNAs with TRBP. The crystal structure of TRBP dsRBD 1 and 2 in complex with a 19 bp siRNA (PDB ID: 5N8L) is available in the RCSB PDB database. We identified the binding residues of TRBP with the control siRNA and docked our designed siRNAs targeting those residues of the structure. We found a number of 8 common interactions between both TRBP‐designed_siRNAs (CagA and VacA) and the control. The interacting residues of control siRNA, designed siRNAs against CagA and VacA with TRBP are shown in Figure 6.

**FIGURE 7:**
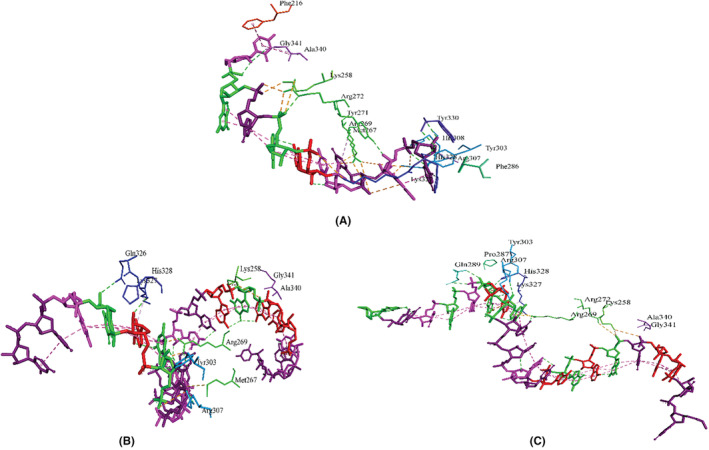
Protein‐siRNA interactions between (A) Argonaute‐2 and control, (B) siRNA_CagA and (C) siRNA_VacA.

Lastly, we performed molecular docking of the guide strands of the two designed siRNAs with human Argonaute‐2. The crystal structure of the human Argonaute‐2 PAZ domain (214–347) in complex with CGUGACUCU (PDB ID: 6RA4) is available in the RCSB PDB database. We identified the binding residues of Agonaute‐2 with the control strand and docked the guide strands from our designed siRNAs targeting those residues of the structure. We found a number of seven common interactions between Argonaute2‐guide strand (CagA) and the control and found nine common interactions between Argonaute2‐guide strand (VacA) and the control. The interacting residues of the control strand, guide strands of the designed siRNAs against CagA and VacA with Argonaute‐2 are shown in Figure 7.

In summary, the constructed siRNAs, whether interacting with TRBP, Dicer, or Ago2, consistently exhibited significantly more negative docking scores (Table 3) compared to their respective control siRNAs, indicating stronger and more stable binding interactions. Particularly, the CagA‐designed siRNA for all three proteins (TRBP, Dicer, and Ago2) stands out with highly negative scores, suggesting strong binding probability. The VacA‐designed siRNA also demonstrated improved binding compared to the controls. Finally, AGO2 protein docking scores reveal both CagA and VacA designed siRNA showcasing higher binding activity compared to the control guide stand.

### 3.5 Molecular dynamics (MD) simulation results

**FIGURE 8:**
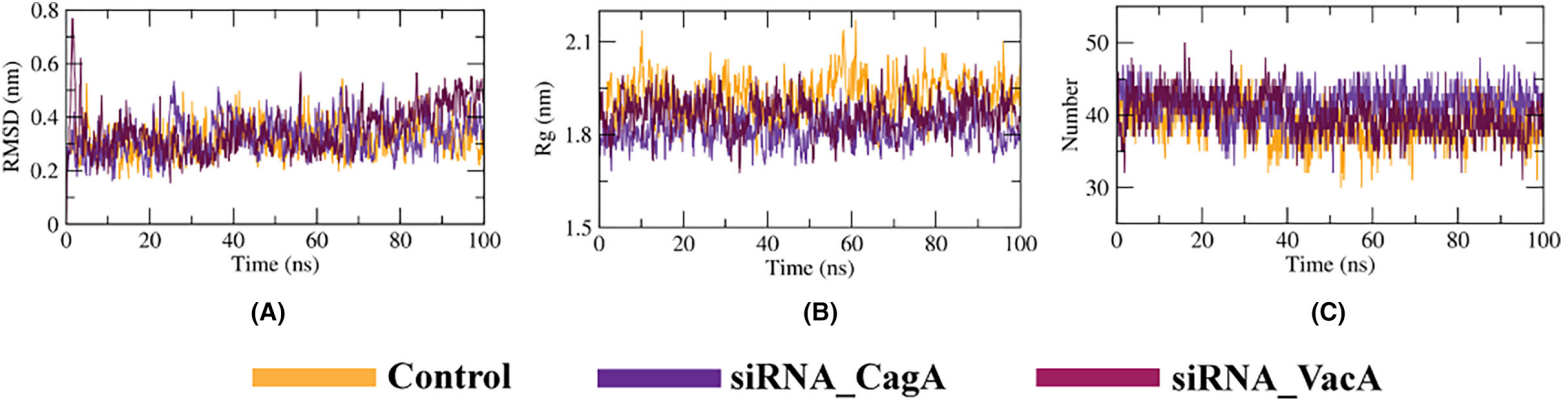
MD simulation results of designed siRNAs. The figure showing (A) RMSD, (B) Rg and (C) hydrogen bonds analysis.

We performed a series of MD simulations of the designed siRNAs and the siRNA‐protein complexes including relevant controls in order to evaluate their atomic level movements. The RMSD plot from the simulation results of the designed siRNAs show that the siRNAs are quite stable and exhibit similar behavior as the control (Figure 8A). The Rg plot showed that the two designed siRNAs were more compact than the control revealed from their lower values (Figure 8B). The number of hydrogen bonds formed during the simulation period was found to be higher than the control (Figure 8C).

**FIGURE 9:**
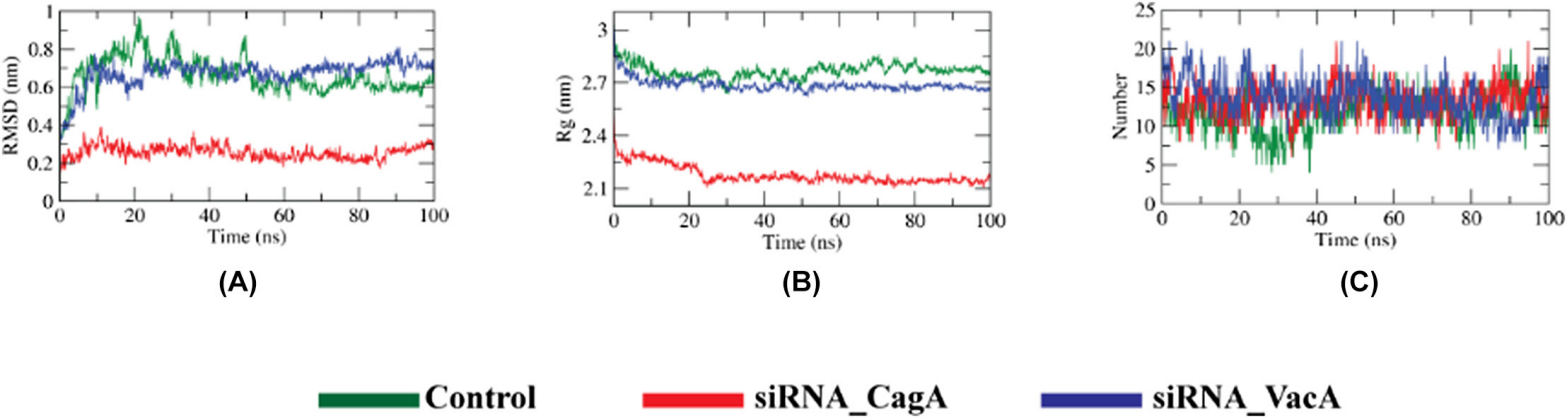
MD simulation results of designed siRNA‐TRBP complexes. The figure showing (A) RMSD, (B) Rg and (C) hydrogen bonds analysis.

The RMSD plot from the simulation results of the designed siRNA‐TRBP complexes depicts that the siRNA‐CagA‐TRBP complex was the most stable while siRNA‐VacA exhibited similar behavior as the control (Figure 9A). The Rg plot showed that the TRBP was more compact in nature while bonded to two designed siRNAs than the control revealed from their lower values (Figure 9B). The number of hydrogen bonds formed during the simulation period was found to be similar compared to the control (Figure 9C).

**FIGURE 10:**
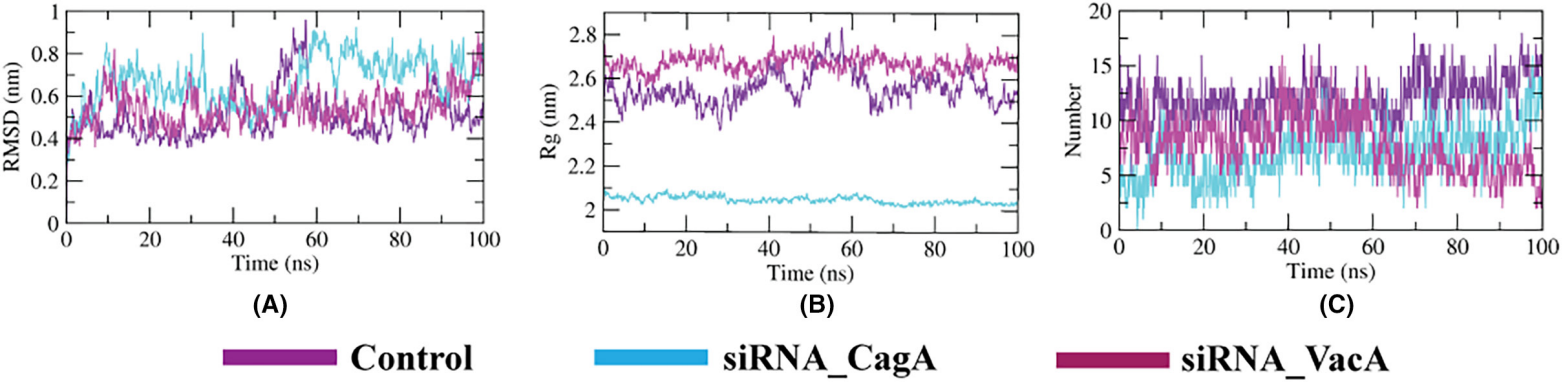
MD simulation results of designed siRNA‐Dicer. The figure showing (A) RMSD, (B) Rg and (C) hydrogen bonds analysis.

The RMSD plot from the simulation results of the designed siRNA‐Dicer complexes showed that the siRNA‐CagA‐Dicer complex showed minor fluctuation throughout the period but the siRNA‐VacA‐Dicer complex exhibited similar behavior as the control (Figure 10A). The Rg plot showed that the dicer was more compact in nature while bonded to two designed siRNAs than the control revealed from their lower values (Figure 10B). The number of hydrogen bonds formed during the simulation period was found to be slightly lower than the control (Figure 10C).

**FIGURE 11:**
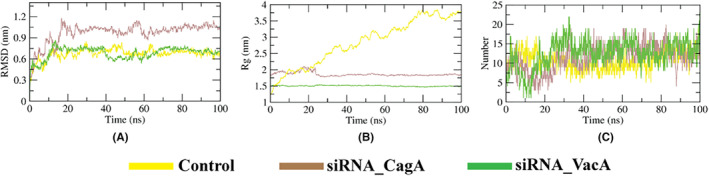
MD simulation results of designed siRNA‐Argonaute‐2 complexes. The figure showing (A) RMSD, (B) Rg and (C) hydrogen bonds analysis.

The RMSD plot from the simulation results of the guide‐Ago2 complexes showed that the guide (CagA)‐Argonaute‐2 complex exhibited a slightly higher value throughout the period but the guide (VacA)‐Argonaute‐2 complex showed similar behavior as the control (Figure 11A). The Rg plot showed that the protein was less compact in nature while bonded to the control strand while the guide strands of the designed siRNAs tend to make the protein more compact revealed from their lower values (Figure 11B). The number of hydrogen bonds formed during the simulation period was found to be similar compared to the control (Figure 11C).

## 4 DISCUSSION

RNA interference (RNAi) is a biological defense mechanism that is used to protect the invasion of various exogenous genes. Small interfering RNA (siRNA) is a promising therapeutic strategy since it has the potential to silence any disease‐related gene in a sequence‐specific manner.^35^ Through the targeted degradation of mRNA or suppression of mRNA translation, siRNA can reduce the expression of target genes, this technology has proved to be promising as therapy in a variety of diseases including cancer.^36^ Our strategy focused on the silencing of *H. pylori*'s key virulence genes CagA and VacA, which are known to play crucial roles in the gastric cancer development.^37^ By designing siRNAs specifically tailored to inhibit the expression of these virulence factors, we aimed to disrupt *H. pylori* pathogenic machinery. Furthermore, through the utilization of nanoparticles such as lipid nanoparticles, gold nanoparticles, PEI and various other techniques, these siRNAs can be introduced into target cells, enabling precise gene knockdown of the gene of interest.^38^

In our current study, we applied a range of criteria to narrow down the search space and enhance the accuracy of siRNA molecule prediction and accuracy. For instance, CagA cDNA sequences collected from seven countries (Bangladesh, Sweden, USA, Australia, China, Philippines, and Vietnam) and VacA cDNA sequences from six countries (Bangladesh, Nepal, Japan, Indonesia, India, and China), we performed multiple sequence alignment (MSA) analysis and the resulting conserved regions of the two genes were used for designing two siRNAs. The two potential siRNAs identified from i‐Score Designer passed the suggested cutoff value for each of the five different algorithms (Ui‐Tei, Amarzguioui, Hsieh, Takasaki, s‐Biopredsi, i‐Score, Reynolds, Ka toh, and DSIR).^26^ The GC content in each of the siRNAs within the 31%–42%, a range considered advisable to be kept between 30% and 60%.^35^ From the thermodynamics analysis, we found that the siRNAs have internal melting temperatures (Tm) below 65°C for both guide and carrier strands. Each siRNA then computed free energies for folding the guide strands ranged from 1.6 to 1.8 and the calculated free energy of binding between the targets and guide strand ranged from −28 to −32 (Table 2). The free energy landscape serves as a valuable indicator of the polycationic binding capability to the nucleic acid and also carries implications for the process of nucleic acid release within the cytosol.^39^

**TABLE 2:** Results of thermodynamic properties of the designed siRNAs.

siRNAs	Melting temperature (Tm)	Energy of antisense strand	Duplex energy
siRNA_CagA‐1	44.6	1.8	−30.2
siRNA_CagA‐2	42.5	1.6	−28.0
siRNA_VacA‐1	46.8	1.8	−32.1
siRNA_VacA‐2	44.6	1.8	−28.8

In molecular docking analysis of a complex, when the energy of the complex is subtracted from its own elements, a low and negative score denotes that the complex is more stable than its individual components alone.^40^ This implies that an exceedingly negative score indicates strong binding, whereas a less negative or even positive score corresponds to a weaker or non‐existent binding. We modeled 3D structures of the finally selected two siRNAs and performed molecular docking with the four major components of the RISC‐Loading complex, TRBP, dicer and the argonaute‐2 proteins. The control siRNAs in these structures were also removed and docked again to compare and validate the docking results. In most cases, the docking scores were notably higher than those of the controls (Table 3). Remarkably, the CagA‐based siRNA consistently demonstrated promising results in each complex. We also found a minimum number of seven common interactions between the designed siRNAs and the respective proteins (Figures 5, 6, 7). To gain deeper insights into the structural dynamics of these complexes, we conducted molecular dynamics simulations (MDS) to study the transport process of siRNA through cell membranes, a powerful computational technique widely employed in the field of structural biology.^41^ As depicted in Figures 8, 9, 10, 11, focused on key parameters indicative of stability and structural behaviors during the MDS period. The root mean square deviation (RMSD) analysis revealed a significant level of stability across all the complexes, with notable low fluctuations. This consistent RMSD pattern underscores the integrity and reliability of our modeled complexes. While the gyration radius, a critical metric in structural biology, provided compelling evidence of the complexes compactness over the course of the simulation,^42^ which highlights the robust and predictable nature of the protein‐siRNAs interactions. An exploration of hydrogen bond formations throughout the MDS yielded further validation of the constructed siRNAs, which signifies strong intermolecular interactions and the maintenance of the complex cohesion. Overall, the employment of MDS in our study adds rigor to our findings and offers a comprehensive understanding of the dynamic behavior of the protein‐siRNA complexes. These results further enhance the credibility and potential utility of our developed siRNAs in targeted gene regulation.

**TABLE 3:** Molecular docking scores of the designed siRNAs with the proteins of the RISC‐Loading complex.

Complex	Docking scores (Kcal/mol)
TRBP‐Control siRNA	−510.62
TRBP‐designed siRNA (CagA)	−794.03
TRBP‐designed siRNA (VacA)	−515.41
Dicer‐Control siRNA	−155.12
Dicer‐designed siRNA (CagA)	−168.42
Dicer‐designed siRNA (VacA)	−142.66
Ago2‐Control guide strand	−152.89
Ago2‐designed siRNA (CagA)	−160.18
Ago2‐designed siRNA (VacA)	−178.09

Moreover, the outcomes of our research necessitate further evaluation to assess the efficacy of the chosen siRNAs in diverse cell lines, whether individually or in combination. Additionally, the exploration of alternative delivery methods and transfection reagents is a priority for future investigations. In summary, our findings highlight the potential of in‐silico siRNA design, selection, filtration, and assessment in advancing the development of next‐generation oligonucleotide‐based therapeutic agents targeted at combatting *H. pylori* infection and protecting the global populations.

## 5 CONCLUSION

It is possible to design and predict siRNA interactions against a specific target gene using computational methods, which will silence that gene's expression. Two siRNA molecules were designed in this study to be effective against the CagA and VacA genes of *H. pylori* using a computational method that took into account all maximum parameters in ideal conditions and cutting‐edge molecular modelling and simulation analyses. The development of therapeutic siRNA techniques could be a potential alternative to decelerate the global gastric cancer cases and recover the affected people.

## AUTHOR CONTRIBUTIONS

**Mahjerin Nasrin Reza:** Conceptualization (equal); data curation (lead); formal analysis (equal); methodology (equal); writing – original draft (lead). **Shahin Mahmud:** Conceptualization (equal); formal analysis (equal); investigation (equal); methodology (equal); supervision (lead); writing – review and editing (equal). **Nadim Ferdous:** Data curation (equal); formal analysis (equal); methodology (equal); writing – original draft (supporting). **Ishtiaque Ahammad:** Data curation (equal); formal analysis (equal); methodology (equal). **Mohammad Uzzal Hossain:** Formal analysis (equal); methodology (equal); validation (equal); writing – review and editing (supporting). **Md. Al Amin:** Formal analysis (equal); methodology (equal); writing – original draft (supporting). **A. K. M. Mohiuddin:** Supervision (supporting); writing – review and editing (equal).

## Data Availability

All data are included in the manuscript and will be available for everyone as per journal policy.

## References

[1] Pellicano R , Ianiro G , Fagoonee S , Settanni CR , Gasbarrini A . Review: extragastric diseases and *Helicobacter pylori* . Helicobacter. 2020;25(S1):19‐25. 10.1111/hel.12741 32918343

[2] Díaz P , Valderrama MV , Bravo J , Quest AFG . *Helicobacter pylori* and gastric cancer: adaptive cellular mechanisms involved in disease progression. Front Microbiol. 2018;9:5. 29403459 10.3389/fmicb.2018.00005 PMC5786524

[3] Farsimadan M , Heravi FS , Emamvirdizadeh A , et al. Evaluation of *Helicobacter pylori* genotypes in obese patients with gastric ulcer, duodenal ulcer, and gastric cancer: an observational study. Dig Dis. 2022;40(3):355‐361. 34010829 10.1159/000517262

[4] Mezmale L , Coelho LG , Bordin D , Leja M . Review: epidemiology of *Helicobacter pylori* . Helicobacter. 2020;40(3):355‐361. 10.1111/hel.12734 32918344

[5] Zagari RM et al. Treatment of *Helicobacter pylori* infection: a clinical practice update. Minerva Med. 2021;112(2):281‐287. 32700868 10.23736/S0026-4806.20.06810-X

[6] Uemura N , Okamoto S , Yamamoto S , et al. *Helicobacter pylori* infection and the development of gastric cancer. N Engl J Med. 2001;345(11):784‐789. 11556297 10.1056/NEJMoa001999

[7] Kumar S , Metz DC , Ellenberg S , Kaplan DE , Goldberg DS . Risk factors and incidence of gastric cancer after detection of *Helicobacter pylori* infection: a large cohort study. Gastroenterology. 2020;158(3):527‐536.e7. 31654635 10.1053/j.gastro.2019.10.019 PMC7010558

[8] Venneman K , Huybrechts I , Gunter MJ , Vandendaele L , Herrero R , van Herck K . The epidemiology of *Helicobacter pylori* infection in Europe and the impact of lifestyle on its natural evolution toward stomach cancer after infection: a systematic review. Helicobacter. 2018;23(3):e12483. 29635869 10.1111/hel.12483

[9] Chalbatani M , Dana H , Gharagouzloo E , et al. Small interfering RNAs (SiRNAs) in cancer therapy: a Nano‐based approach. Int J Nanomedicine. 2019;14:3111‐3128. 31118626 10.2147/IJN.S200253 PMC6504672

[10] Kalita T , Dezfouli SA , Pandey LM , Uludag H . SiRNA functionalized lipid nanoparticles (LNPs) in management of diseases. Pharmaceutics. 2022;14(11):1‐35. 10.3390/pharmaceutics14112520 PMC9694336 36432711

[11] Zhang X , Goel V , Robbie GJ . Pharmacokinetics of Patisiran, the first approved RNA interference therapy in patients with hereditary transthyretin‐mediated amyloidosis. J Clin Pharm. 2019;60:573‐585. 10.1002/jcph.1553 PMC7187331 31777097

[12] Dobrowolski C , Paunovska K , Hatit MZC , Lokugamage MP , Dahlman JE . Therapeutic RNA delivery for COVID and other diseases. Adv Healthc Mater. 2021;10(15):e2002022. 33661555 10.1002/adhm.202002022 PMC7995096

[13] Kim YK . RNA therapy: rich history, various applications and unlimited future prospects. Exp Mol Med. 2022;54(4):455‐465. 35440755 10.1038/s12276-022-00757-5 PMC9016686

[14] Sohrab SS , Aly el‐Kafrawy S , Mirza Z , Hassan AM , Alsaqaf F , Azhar EI . In Silico prediction and experimental validation of SiRNAs targeting ORF1ab of MERS‐CoV in Vero cell line. Saudi Journal of Biological Sciences. 2021;28(2):1348‐1355. 33519276 10.1016/j.sjbs.2020.11.066 PMC7833792

[15] Oany AR , Hossain MU , Ahmad SAI . Computational approach to design a potential SiRNA molecule to silence the Nucleocapsid gene of different Nipah virus strains of Bangladesh. Biores Comm. 2015;1(1):40‐44.

[16] Chowdhury UF , Sharif Shohan MU , Hoque KI , Beg MA , Sharif Siam MK , Moni MA . A computational approach to design potential SiRNA molecules as a prospective tool for silencing nucleocapsid phosphoprotein and surface glycoprotein gene of SARS‐CoV‐2. Genomics. 2021;113(1 Pt 1):331‐343. 33321203 10.1016/j.ygeno.2020.12.021 PMC7832576

[17] Idris A , Davis A , Supramaniam A , et al. A SARS‐CoV‐2 targeted SiRNA‐nanoparticle therapy for COVID‐19. Mol Ther. 2021;29(7):2219‐2226. 33992805 10.1016/j.ymthe.2021.05.004 PMC8118699

[18] Evers MJW , van de Wakker SI , de Groot EM , et al. Functional siRNA delivery by extracellular vesicle–liposome hybrid nanoparticles. Adv Healthc Mater. 2022;11(5):e2101202. 34382360 10.1002/adhm.202101202 PMC11468224

[19] Gupta N , Rai DB , Jangid AK , Pooja D , Kulhari H , et al. Nanomaterials‐based SiRNA delivery: routes of administration, hurdles and role of nanocarriers. Nanotechnology in Modern Animal Biotechnology. 2019;2019:67‐114.

[20] Cover TL . *Helicobacter pylori* diversity and gastric cancer risk. mBio. 2016;7(1):1‐9. 10.1128/mBio.01869-15 PMC4742704 26814181

[21] Nejati S , Karkhah A , Darvish H , Validi M , Ebrahimpour S , Nouri HR . Influence of *Helicobacter pylori* virulence factors CagA and VacA on pathogenesis of gastrointestinal disorders. Microb Pathog. 2018;117:43‐48. 29432909 10.1016/j.micpath.2018.02.016

[22] Mucito‐Varela E , Castillo‐Rojas G , Calva JJ , López‐Vidal Y . Integrative and conjugative elements of *Helicobacter pylori* are hypothetical virulence factors associated with gastric cancer. Front Cell Infect Microbiology. 2020;10:1‐14. 10.3389/fcimb.2020.525335 PMC7604443 33194783

[23] Alipour M . Molecular mechanism of *Helicobacter pylori*‐induced gastric cancer. J Gastrointest Cancer. 2021;52(1):23‐30. 32926335 10.1007/s12029-020-00518-5 PMC7487264

[24] Wang D , Guo Q , Yuan Y , Gong Y . The antibiotic resistance of *Helicobacter pylori* to five antibiotics and influencing factors in an area of China with a high risk of gastric cancer. BMC Microbiol. 2019;19(1):152. 31272365 10.1186/s12866-019-1517-4 PMC6611032

[25] Sievers F , Higgins DG . Clustal Omega. Curr Protoc Bioinformatics. 2014;48(1):3‐13. 10.1002/0471250953.bi0313s48 25501942

[26] Ichihara M , Murakumo Y , Masuda A , et al. Thermodynamic instability of SiRNA duplex is a prerequisite for dependable prediction of SiRNA activities. Nucleic Acids Res. 2007;35(18):e123. 17884914 10.1093/nar/gkm699 PMC2094068

[27] Wu Y , Shi B , Ding X , et al. Improved prediction of RNA secondary structure by integrating the free energy model with restraints derived from experimental probing data. Nucleic Acids Res. 2015;43(15):7247‐7259. 26170232 10.1093/nar/gkv706 PMC4551937

[28] Wenzel A , Akbaşli E , Gorodkin J . RIsearch: fast RNA‐RNA interaction search using a simplified nearest‐neighbor energy model. Bioinformatics. 2012;28(21):2738‐2746. 22923300 10.1093/bioinformatics/bts519 PMC3476332

[29] Popenda M , Szachniuk M , Antczak M , et al. Automated 3D structure composition for large RNAs. Nucleic Acids Res. 2012;40(14):e112. 22539264 10.1093/nar/gks339 PMC3413140

[30] Bernhart SH , Hofacker IL , Will S , Gruber AR , Stadler PF . RNAalifold: improved consensus structure prediction for RNA alignments. BMC Bioinformatics. 2008;9:1‐13. 19014431 10.1186/1471-2105-9-474 PMC2621365

[31] Burley SK , Bhikadiya C , Bi C , et al. RCSB protein data Bank: powerful new tools for exploring 3D structures of biological macromolecules for basic and applied research and education in fundamental biology, biomedicine, biotechnology, bioengineering and energy sciences. Nucleic Acids Res. 2021;49:D437‐D451. 33211854 10.1093/nar/gkaa1038 PMC7779003

[32] Yan Y , Zhang D , Zhou P , Li B , Huang S‐Y . HDOCK: a web server for protein‐protein and protein‐DNA/RNA docking based on a hybrid strategy. Nucleic Acids Res. 2017;45(W1):W365‐W373. 28521030 10.1093/nar/gkx407 PMC5793843

[33] Studio D . Dassault Systemes BIOVIA, Discovery Studio Modelling Environment, Release 4.5. Accelrys Software Inc.; 2015.

[34] Abraham MJ , Murtola T , Schulz R , et al. Gromacs: high performance molecular simulations through multi‐level parallelism from laptops to supercomputers. SoftwareX. 2015;1‐2:1‐2.

[35] Friedrich M , Aigner A . Therapeutic SiRNA: state‐of‐the‐art and future perspectives. BioDrugs. 2022;36(5):549‐571. 35997897 10.1007/s40259-022-00549-3 PMC9396607

[36] Kandasamy G , Maity D . Current advancements in self‐assembling nanocarriers‐based SiRNA delivery for cancer therapy. Colloids and Surfaces B: Biointerfaces. 2023;221:226‐237. 10.1016/j.colsurfb.2022.113002 36370645

[37] Wang B , Gan Q , Tong Y , et al. A visual diagnostic detection of *Helicobacter pylori* and the gastric carcinoma‐related virulence genes (CagA and VacA) by a fluorescent loop‐mediated isothermal amplification (LAMP). Talanta. 2023;256:124260. 36640706 10.1016/j.talanta.2023.124260

[38] Paul A , Muralidharan A , Biswas A , Kamath BV , Joseph A , Alex AT . siRNA therapeutics and its challenges: recent advances in effective delivery for cancer therapy. OpenNano. 2022;7:100063.

[39] Grasso G , Deriu MA , Patrulea V , Borchard G , Möller M , Danani A . Free energy landscape of SiRNA‐Polycation complexation: elucidating the effect of molecular geometry, polymer flexibility, and charge neutralization. PLoS One. 2017;12(10):e0186816. 29088239 10.1371/journal.pone.0186816 PMC5663398

[40] Fan J , Ailing F , Zhang L . Progress in molecular docking. Quantitative Biology. 2019;7(2):83‐89.

[41] Rouhani M , Khodabakhsh F , Norouzian D , Cohan RA , Valizadeh V . Molecular dynamics simulation for rational protein engineering: present and future prospectus. J Mol Graph Model. 2018;84:43‐53. 29909273 10.1016/j.jmgm.2018.06.009

[42] Lobanov MI u , Bogatyreva NS , Galzitskaya OV . Radius of gyration as an indicator of protein structure compactness. Mol Biol. 2008;42(4):701‐706. 18856071

